# A proposal for ranking through selective computation of centrality measures

**DOI:** 10.1371/journal.pone.0289488

**Published:** 2023-09-18

**Authors:** Daniele Bertaccini, Alessandro Filippo

**Affiliations:** Department of Mathematics, University of Rome Tor Vergata, Rome, Italy; Universidad Diego Portales, CHILE

## Abstract

In complex network analysis it is essential to investigate the alteration of network structures that results from the targeted removal of vertices or edges, ranked by centrality measures. Unfortunately, a sequential recalculation of centralities after each node elimination is often impractical for large networks, and computing rankings only at the beginning often does not accurately reflect the actual scenario. Here we propose a first result on the computational complexity of the sequential approach when nodes are removed from a network according to some centrality measures based on matrix functions. Moreover, we present two strategies that aim to reduce the computational impact of the sequential computation of centralities and provide theoretical results in support. Finally, we provide an application of our claims to the robustness of some synthetic and real-world networks.

## 1 Introduction

One of the most challenging problems in network analysis is identifying the most vital nodes for the correct functioning of an interconnected system [1]. These special nodes are essential for fast information spreading in social and technological networks as well as in structural resilience of infrastructural networks [2]. Consequently, the demand of a reliable metric that quantify the importance of a node has given rise to a highly popular area of research, resulting in the introduction of various definitions of *node centrality* [3] and other analogous tools; see [4, 5]. Among the possible *centrality measures*, we will concentrate on those based on matrix functions of the adjacency matrix because of their ability to take into account certain communicability characteristics of the network; see, e.g., [6].

A major issues with centrality measures is that unexpected structural changes, which frequently occur in modern evolving networks, can quickly make our understanding of the most critical components obsolete. For example, crucial nodes or connections may disappear as a result of either a random failure, or a targeted attack in which an external agent deliberately damages the system.

In previous works [7, 8], two approaches were basically proposed: the simultaneous strategy, consisting of considering the centrality measures as constants after the elimination, or the sequential one, consisting of computing the centrality measures each time an element is removed from the system. Both strategies have pros and cons and some studies address the difference of these two methods proving that often one is more suitable than the other depending on the situation. For instance, in attack robustness of complex networks the analysis of the sequential approach resulted to be crucial to obtain the best results; see [9] and the recent survey [10] for more details. On the other hand, in problems such as optimization of network communicability via edge removal, recomputing the centrality measures was demonstrated to be not of fundamental importance [11].

Here we are interested in frameworks where recomputing certain centrality measures can have a significant impact on the result. Consequently, we tried to give an answer to the question: since recomputing the centrality measure after network modifications can be a computationally expensive operation, is it possible to reduce the computations with a ranking comparable with the sequential approach?

Our proposals, based on what we named *selective computation*, are based on the idea that there exist some indicators that suggest when the network is so changed that it is necessary to recompute the centrality measure. This work finds theoretical motivation in the study of centrality measures variations in evolving networks [12]. Related works concentrated on finding bounds of walk based centrality measures after modification of the adjacency matrix [13]. Other approaches included proposing ad-hoc *dynamic centrality measures* to formally define the importance of a node in temporal networks [14].

We provide an application of our claims to sequential network attacks. Other examples of application include the optimization of communicability via node removal (see, e.g., [11]) and the fast identification of crucial nodes in dynamic networks (see, e.g., [14]).

In Section 2 we briefly recall some basics on complex networks. Some definitions about centrality measures are reported in Section 3. In Section 4 some issues and computational cost of simultaneous and sequential attacks are discussed. Then, our new strategies of threshold and correlation attacks are introduced in Section 5. Section 6 and 7 are dedicated to numeric experiments and to an application to network robustness. Finally, some concluding remarks and future directions are reported in Section 8.

## 2 Main background

A well known way to represent the interactions in a network is through the use of graph models; see, e.g., [1, 3] and references therein. A *graph* *G* is defined by a set of *nodes* (or *vertices*) *V* = {*v*_1_, …, *v*_*n*_} and a set of *edges* *E* ⊆ *V* × *V*. *G* = (*V*, *E*) is *undirected* if (*v*_*i*_, *v*_*j*_) ∈ *E* implies (*v*_*j*_, *v*_*i*_) ∈ *E*, otherwise *G* is *directed*.

A *walk* of length *l* is a sequence of *l* edges which joins a sequence of vertices in *V*. If all vertices (and thus all edges) in a walk are distinct we call it a *path*. An undirected graph is *connected* if for each distinct pairs of nodes there is a walk between them. A directed graph is *strongly connected* if for each distinct pairs of nodes *v*_*i*_, *v*_*j*_, there is a direct walk from *v*_*i*_ to *v*_*j*_. More details can be found in [1, 3].

For a directed and an undirected graph *G*, considered here unweighted for simplicity of notation, we introduce the *adjacency matrix* *A* as the *n* × *n* matrix with entries

$$\begin{array}{c} {\left(A\right)}_{i,j}=a_{ij}=\{\begin{array}{cc} 1 & \;\text{if}\;(v_{i},v_{j})\in E,\\ 0 & \;\text{otherwise}. \end{array} \end{array}$$

Here we will concentrate on undirected networks which are represented by graph having symmetric adjacency matrices, but most of the discussions can be extended to directed as well. Moreover, we will consider only networks corresponding to *simple graphs* that do not contain *loops* (edges from a node to itself) or multiple edges.

The importance of an element in an interconnected system is typically referred to as its *centrality* [1]. Over the years, a wide variety of measures have been proposed to quantify the centrality of a node, with each focusing on different concepts; see, e.g., [3] for more details and [15] for recent examples. Some centrality measures, which will be recalled in the next section, are defined using functions of the adjacency matrix such as the exponential and the resolvent function; see, e.g., [6].

One of the most studied topics in network science, and somewhat related to the problem of quantifying the centrality of a node, is investigating the effect on the network structures when removing its components such as nodes or edges. For instance in power grid networks, it is important to study the potential issues that may occur after a malfunction [16]. Other examples include breakdowns of communication networks [17] and disruptions of biological systems [18].

Various works, such as [19], focus on the consequences of systems random failures modeled by uniform random elimination of nodes. This is the most extensively studied case due to its well known connections with percolation theory [20]. Other studies are instead on targeted attacks to the network structures, which consist of eliminating nodes following a specific list. For example, one can remove nodes in order of degree [2] or other centrality measures such as betweenness, closeness and eigenvector centrality [9]. Recent studies proposed efficient and effective ways to degrade a network based on strategies that do not strictly involve centrality measures [4, 21].

In addition to studying implications of node removal from a network, it is also possible to consider attacks based on targeted removal of arcs. The choice between the two methods depends exclusively on the context. In a dynamical complex network like a social network, more often it is people, rather than relationships, which are removed from a network. On the other hand, in a technological or infrastructural network, the failure of connections or information channels should also be taken into consideration; the only difference lies in the chosen definition of *edge centrality*. An example of such an investigation is present in [7], where the arcs are removed in descending order according to their *edge betweenness centrality*, which is defined as the proportion of the shortest paths that traverse that arc, and on the basis of a possible definition of *edge degree centrality*, given by the product of the degrees of the nodes at the edges’ extremes.

## 3 Some centrality measures

For every node *v*_*i*_ ∈ *V* the first centrality index we mention is the *degree* *d*_*i*_, which is, for undirected networks, the number of edges in *E* leaving *or* entering *v*_*i*_. In terms of the adjacency matrix *A*, *d*_*i*_ can be written as:

$$\begin{array}{c} d_{i}=\sum_{j=1}^{n}\;a_{ij}={\left[A1\right]}_{i}, \end{array}$$

where **1** is the vector of all ones in $R^{n}$. Here we concentrate mostly on *total communicability* introduced in [22] and on *subgraph centrality* introduced in [23], but the discussion can be extended to other walk based indices that can be computed as functions of the adjacency matrix such as those in, e.g., [6]. These measures are interesting as they can detect a node global influence by looking at the walks (with a suitable weight) in which the node is involved, balancing long and short range connections by giving greater emphasis on short walks rather than long ones. In addition, total communicability benefits of being computationally more efficient than other nonlocal centrality measures in the case of large networks, which has proven to be essential in some practical application such as [24].

For the *i*−th node of a network with adjacency matrix *A*, total communicability or *TC*_*i*_(*A*) for short, is defined using the exponential of a matrix

$$e^{A}=I+A+\frac{A^{2}}{2!}+\cdots =\sum_{k=0}^{\infty }\frac{A^{k}}{k!},$$

i.e.

$$\begin{array}{c} TC_{i}\left(A\right)=\sum_{j=1}^{n}{\left[e^{A}\right]}_{ij}={\left[e^{A}1\right]}_{i}, \end{array}$$ (1)

while the subgraph centrality of node *i*, *SC*_*i*_(*A*) for short, is given by

$$\begin{array}{c} SC_{i}\left(A\right)={\left[e^{A}\right]}_{ii}, \end{array}$$

i.e., the *i*-th diagonal entry of *e*^*A*^, see [22, 23] for more details.

Notice that from medium size network on, it is of paramount importance to use techniques for sparse matrices. Indeed, adjacency matrices for complex networks are usually very sparse, i.e., their number of nonzero entries are of the order of the number of row or columns. In order to implement efficiently the underlying computations, a parallel environment is also frequently considered when the network is huge (e.g., Google, Facebook, etc.). Moreover, fast computation of some centrality indices require evaluating *f*(*A*)**v** for a certain vector **v**, including total communicability, and some of the related techniques require solving large linear systems; see discussions in [25]. This is a computationally delicate matter; see, e.g., [26] for details.

## 4 Simultaneous and sequential attacks: Issues and (computational) cost

As noted in Section 2, one way to choose the nodes to hit with a targeted attack is to consider the order given by the centrality measures which, by identifying the most influential nodes in a network, can also identifies its weak points.

Suppose we selected a measure of centrality and want to perform a targeted attack by removing a specific fraction of the nodes from a network, for example, equal to 1% of the total. In that case, the choice of nodes to hit would fall on the “best” 1% of the nodes in terms of ranking, which are the 1% of the nodes that have the highest centrality indices. However, with the same fraction of nodes removed from a system and the same centrality measure selected, two different approaches to pursuing an attack on a network are possible. In the first, the removal of the nodes is carried out in descending order of importance according to the ranking calculated *before* the attack begins, this until the desired percentage is reached. This are usually called *simultaneous attacks* on a network see, e.g., [9], and, in some situations, it represents the natural pattern to proceed. In the context of a vaccination campaign, for example, it is reasonable to consider each contact as having occurred at the same time and proceed with vaccination prioritizing individuals based on the risk factor(s) they had at the start of the vaccination campaign. In the second basic approach, the removal of the nodes is performed in sequence, recalculating the centrality indices each time a change is made within the network, that is usually called *sequential attack*; see, e.g., [9].

In most cases, sequential attacks are a natural method for node removal. A typical case can be found in investigating how the failure of a router in an Internet connection can affects the others in a cascade failure. Another crucial example is the elimination of nodes from an organism’s protein-protein interaction net in which the mutations that make a protein biologically inactive (unable to interact with its partner proteins) compromise in succession all the protein patterns in the organism. The above are just two examples of networks that should be modelled by sequential attacks and not by a paradigm based on simultaneous attacks [9].

In general, simultaneous and sequential attacks can produce very different effects on the same network, even when the number of removed nodes is relatively small. This is because the sequence of node elimination may vary significantly between the two strategies, resulting in two different rankings.

**Fig 1:**
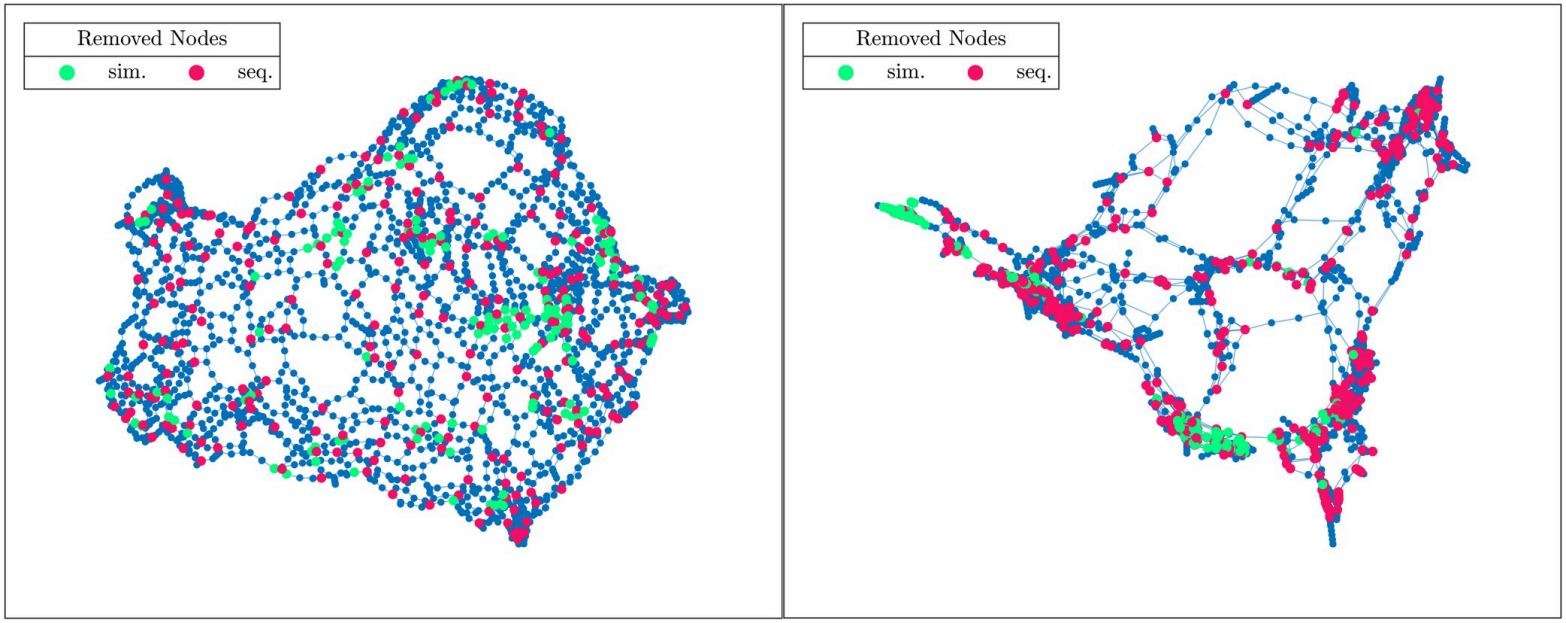
Simultaneous and sequential attacks for *Minnesota* and *Power* networks. Nodes removed in the networks *Minnesota* (on the left) and *Power* (on the right), in the case of simultaneous (in green) and sequential (in red) attacks. The images are related to the cases in which the top 10% of the nodes are removed according to TC.

One of the ways to compare two rankings in, e.g., two ordered lists, is to consider the *intersection distance* (or *intersection similarity*). The latter compares two sorted lists with the following formula [27]:

$$\begin{array}{c} isim_{k}\left(x,y\right)=\frac{1}{k}\sum_{i=1}^{k}\frac{|x_{i}\Delta y_{i}|}{2i}, \end{array}$$ (2)

where Δ is the symmetric difference operator between two sets, *x*_*i*_ and *y*_*i*_ represent the first *i* elements in the sorted lists *x* and *y*. Two identical lists will lead to having *isim*_*k*_(*x*, *y*) = 0 for each *k* while two different lists will give *isim*_*k*_(*x*, *y*) approaching one. In Section 6 we compare the intersection distances between the rankings obtained with simultaneous and sequential attacks (as well as our proposed strategies) for some test cases. One can get an idea and see how much the discrepancy can be by looking at Fig 1, which highlights in different colours the top 10% of nodes according to TC, removed from the *Minnesota* and *Power* networks (both available in [28]) by the two strategies.

As a result of this discrepancy, simultaneous attacks may not be accurate enough. Therefore, it is often preferable to use sequential attacks, despite they can require more computational resources for most centrality measures and surely for those based on matrix functions. The computational cost of sequential attacks does, in fact, increase with the number of removed nodes, even if the process becomes less computationally demanding as the network size decreases. In the following, we give a simple idea of the computational cost of sequential attacks based on the chosen type of centrality, the size of the network and the percentage of the removed nodes.

Let $f:R^{n\times n}\to R^{n}$ be a centrality measure whose calculation requires only products of the adjacency matrix per vector plus operations of lower computational complexity (this is the case of TC) and let *G* be a sparse graph, that is a graph of order *n* whose number of edges is *O*(*n*). Assuming that the computation of *f*(*A*), A adjacency matrix of *G*, can be carried out with *O*(*n*) operations and that this is the most expensive operation of the procedure (not counting the cost to find the maximum and remove nodes from the graph).

**Proposition 4.1**. *The number of operations required to assess the impact of a sequential attack on a sparse graph with n nodes in which a fraction δ* ∈ (0, 1) *of them is removed, according to the ranking given by f, is of the order of at most*

$$Cn^{2}\hat{\delta }+Cn(1-\frac{\hat{\delta }}{2}),$$

*where C is a constant that depends on the sparsity of the graph and* $\hat{\delta }$ *is chosen so that* $\hat{\delta }n$ *is the integer immediately greater than or equal to δn*.

*Proof*. If *G* is a sparse matrix, *G* can be assumed to remain sparse during the node removal process. This is true since the removal of a node is always equivalent to the removal of at least one arc, especially if the node has high degree. This means that considering *C* as a constant is a conservative estimate on the degree of sparsity of the graph. Chosen $\hat{\delta }$ so that $\hat{\delta }n=\left[\delta n\right]+1$, where [*x*] denotes the integer part of *x*, the computational cost to compute *f* $\hat{\delta }n$ times is

$$\begin{array}{cc} \sum_{i=0}^{\hat{\delta }n}\;C\left(n-i\right) & =Cn\left(\hat{\delta }n+1\right)-\sum_{i=0}^{\hat{\delta }n}iC\\ & =Cn^{2}\hat{\delta }+Cn-\frac{\hat{\delta }n\left(\hat{\delta }n+1\right)}{2}C\\ & =Cn^{2}(\hat{\delta }-\frac{{\hat{\delta }}^{2}}{2})+Cn(1-\frac{\hat{\delta }}{2})\\ & \approx Cn^{2}\hat{\delta }+Cn(1-\frac{\hat{\delta }}{2}), \end{array}$$

where in the last step we neglected the second order term in $\hat{\delta }$.

From Proposition 4.1, it is possible to observe that if *δ* tends to zero, the computational cost tends to that of simultaneous attacks, or rather to the cost of performing the calculation of the centrality index once. On the other hand, if *δ* tends to 1, the cost will approach *O*(*n*^2^).

## 5 Selective computation of centrality measures

At the core of our proposals, devised to reduce the computational cost of sequential attacks for certain centrality measures, is the notion of *selective computation*. The latter consists in recomputing a centrality measure only when specific conditions are met, potentially obtaining similar results of sequential attacks with less computational effort. Indeed, simultaneous attacks consider the centrality measures as constants over time, often a poor approximation of reality as they can rapidly change with node (or arc) elimination. Consequently, nodes initially considered crucial in the network can rapidly loose their importance. Using sequential attacks solve this problem by constantly updating our understanding of the network, but this, as observed in the previous section, can require more computational resources.

The first strategy is based on the lost quantity of normalized centrality and recompute centralities only when predetermined thresholds are met. We call it *threshold attack(s)*. The second one is called *correlation attack(s)* and exploits the correlation among the centralities values of neighboring nodes. In each of these two latter cases, we can say that the ranking calculated at the beginning is “selectively updated”.

### 5.1 Threshold attacks

The obvious reason behind the high computational cost of sequential attacks is that the centrality measures are computed every time a node (or an arc) is removed from the system. However, the impact of a node (or arc) removal on the network is not the same. The more significant the node (arc) is, the higher the chances of a drastic alteration in the network. Therefore, a reasonable strategy to replace sequential attacks can be to recompute the centrality measures only after eliminating a node (arc) with relatively high centrality.

Our approach consists of first computing the vector of the centrality measures **c**, normalized by the sum of its entries, and then removing from the network the first nodes that appear in the ranking until the sum of centralities of the removed nodes is greater than a selected threshold. Then we recompute the measures repeating the process until we remove the desired quantity of nodes from the network.

Algorithm 1 outlines the details of the threshold attacks technique. We will denote by (*A*)_*i*,:_ the *i*-th row of the adjacency matrix *A*, by (*A*)_:,*i*_ the *i*-th column of *A* and by **0** the zero vector of $R^{n}$.

**Algorithm 1. Threshold attacks**

**Input**: $A\in R^{n\times n}$ adjacency matrix of *G*, $M\in N_{>0}$ number of nodes to be removed, *γ* ∈ (0, 1) threshold value.

**Output**: Set S of *M* nodes removed from *G*.

1: S=∅;

2: **for** *m* = 1, …, *M* **do**

3:  **if** *m* == 1 or else *flag* == 1 **then**

4:   compute the centrality vector of interest **c**;

5:   $sum=\sum_{i=1}^{n}c_{i}$;

6:   **c** = **c**/*sum*;

7:  **end if**

8:  $i^{*}=\underset{i}{\text{argmax}}\;c_{i}$;

9:  (*A*)_i*,:_ = **0**^*T*^;

10:  (*A*)_:,i*_ = **0**;

11:  **c**_i*_ = 0;

12:  $flag=sum-\sum_{i=1}^{n}c_{i}>\gamma$;

13:  $S=S\cup \{i^{*}\}$;

14: **end for**

Note that the more computationally expensive operation of Algorithm 1 is the computation of the centrality measure **c** on line 4. Consequently, we can estimate the computational cost of Algorithm 1 by the number of **c** computations, which can be much less than the number of nodes eliminated, *M*, depending on the threshold value. Section 6 provides further details on how to choose a suitable threshold for a given network.

Finally, it is worth mentioning that threshold attacks can be considered as a generalization of the sequential strategy when the threshold value is set to zero.

### 5.2 Correlation attacks

The main idea is exploiting the correlation among the centrality values of neighboring nodes in walk-based centrality measures like *subgraph centrality* and *total communicability*. For example, a node adjacent to a critical node takes much of its importance. Most of the short walks starting from it, which are crucial for its centrality, pass through that particular connection. Therefore, after the targeted removal of that crucial node, the adjacent node will suffer from a drop in the value of its centrality measure. On the other hand, the centrality measures of two distant nodes can be more weakly correlated because the fading of one of the two influences only long walks between them, inducing a slight decrease in the centrality value of the other.

**Proposition 5.1**. *If i and j are two nodes of the unweighted and undirected graph G with dist*(*i*, *j*) = *s* > 0, *then*

a *All the walks of length less than s that start from j are preserved after removing i from G*.
b *If* $\hat{TC}\left(j\right)$ *is the total communicability of the node j after the removal of i from G, then*
$$\begin{array}{c} \hat{TC}\left(j\right)=TC\left(j\right)-\sum_{k=s}^{\infty }\frac{C_{k}\left(i,j\right)}{k!}, \end{array}$$ (3)
  *where* *TC*(*j*) *is the total communicability of j before the removal of i and C*_*k*_(*i*, *j*) *is the number of walks of length k that start form j and pass through the node i*.
c *If* $\epsilon \left(j\right)=|TC\left(j\right)-\hat{TC}\left(j\right)|$ *is the drop of total communicability faced by j after the removal of i from G, then*
$$\begin{array}{c} \epsilon (j)\to 0\;\text{when}\;s\to \infty . \end{array}$$

*Proof*. (a.) If *dist*(*i*, *j*) = *s* > 1, then the length of the shortest path between *i* and *j* is *s*; this means that every walk that starts from *j* with a length less than *s* can not be affected by the removal of node *i*. (b.) By definition, *TC*(*j*) is equal to the sum of all walks that start from *j*, weighing by their length *k* by a penalization factor of 1/*k*!; see [22]. Among those walks, some of them start from *j* and pass trough *i*; this means that they will disappear after the removal of *i* from *G*. Let *C*_*k*_(*i*, *j*) be the number of lost walks of length *k* ≥ 1; we can derive $\hat{TC}\left(j\right)$ by summing over all possible lengths *k*, remembering that for (a.) *C*_*k*_(*i*, *j*) = 0 for all *k* < *s*. Note that if *G* is a complete graph with *n* nodes, then *C*_*k*_(*i*, *j*) = *n*^(*k*−1)^ because a walk of length *k* that pass through *i* is a collection of nodes {*j*, *v*_2_, …, *v*_*k*+1_} where *v*_*l*_ = *i* for at least one *l* = 2, …, *k* + 1. So, the fact that *C*_*k*_(*i*, *j*) ≤ *n*^(*k* − 1)^ for all *k* ≥ 1 ensures that the series in (3) converges. (c.) By definition, *ϵ*(*j*) is the tail of a convergent series; so the convergence of the series implies *ϵ*(*j*) → 0.

Note that Proposition 5.1 implies that if *s* → ∞, i.e. the node *i* is far from the node *j*, the value of *TC*(*j*) does not change much after the removal of *i*, confirming the intuition that removing a node influences the centrality measures of nearby nodes more than the centrality indices of those that are more far away. Moreover, if *i* and *j* belongs to two different connected components, i.e. *dist*(*i*, *j*) = +∞, *TC*(*j*) does not change at all because *C*_*k*_(*i*, *j*) = 0 for all *k* ≥ 1. Remarkably this can be seen as a generalization of what happens with node degree, where the elimination of a node only alters the degree of adjacent nodes.

**Remark 5.1**. *Proposition 5.1 can be generalized to take account other walk-based centrality measures such as* subgraph centrality *by replacing* *C*_*k*_(*i*, *j*) *into the number of closed walks of length* *k* *that start form* *j* *and pass trough* *i*.

We now discuss the strategy of correlation attacks based on Proposition 5.1. Our scheme consists of first computing the centrality ranking while removing the first node and then recomputing the centrality measures only if the following node in the ranking is adjacent to the removed one (see Remark 5.3 for nodes connected by walks). After *M* steps, we have successfully removed *M* nodes from the network by doing a few recalculations of the centrality measures, potentially saving computational time. This approach should work as expected because while during an attack procedure, the ranking obtained at the beginning changes after removing the first node, it can still be considered a good approximation depending on which node lies in second position. For example, if the next node in the ranking is far from the first, its centrality measure should remain approximately the same. So, it is reasonable to assume that its position in the ranking will not change much because only a slight decrease in its centrality measure is expected.

Algorithm 2 contains a description of correlation attacks technique. The notation is the same used in Algorithm 1.

**Algorithm 2**. **Correlation attacks**

**Input**: $A\in R^{n\times n}$ adjacency matrix of *G*, $M\in N_{>0}$ number of nodes to be removed.

**Output**: Set S of *M* nodes removed from *G*.

1: S=∅;

2: **for** *m* = 1, …, *M* **do**

3:  **if** *m* == 1 or else *adjacent* == 1 **then**

4:   compute the centrality vector of interest **c**;

5:  **end if**

6:  $i^{*}=\underset{i}{\text{argmax}}\;c_{i}$;

7:  $j^{*}=\underset{j,j\neq i^{*}}{\text{argmax}}\;c_{j}$;

8:  *adjacent* = (*A*)_i*, j*_ > 0;

9:  (*A*)_i*,:_ = **0**^*T*^;

10:  (*A*)_:,i*_ = **0**;

11:  **c**_i*_ = 0;

12:  $S=S\cup \{i^{*}\}$;

13: **end for**

It should be noted that, since the most expensive operation of Algorithm 2 is the computation of the centrality measure **c** on line 4, the majority of the computational cost of correlation attacks is given by the number of times that **c** is computed from scratch (exactly as Algorithm 1). Clearly this number of recalculation is dependent on the network. However, we can say whether the number of recalculations will fall strictly between one and *M* (see Remark 5.2).

**Remark 5.2**. *At the first step of the Algorithm 2, let* $\bar{V}=\left\{v_{\alpha_{1}},\ldots ,v_{\alpha_{n}}\right\}$ *be the nodes of G ordered by their centrality measures and let* $\bar{A}$ *be the matrix obtained from* *A* *by permuting its rows (and columns) following the order* {*α*_1_, …*α*_*n*_}. *If* $U_{M}=\left\{{\left(\bar{A}\right)}_{i,i+1}:i=1,\ldots ,M\right\}$ *are the first* *M* *elements of the upper diagonal of* $\bar{A}$, *then Algorithm 2 computes the centrality measures at least more than once if there is at least one non zero element in* *U*_*k*_. *Moreover, if* m* *is the smallest* *i* *such that* ${\left(\bar{A}\right)}_{i,i+1}>0$ *then Algorithm 2 computes the centrality measure no more than* *M* + 1 − m* *times*.

**Remark 5.3**. *It is possible to refine Algorithm 2 by considering generic walks of length* *l* ≥ 2 *between the first two nodes in the ranking. It is only necessary to change line 8 with* (*A*)_i*,j*_ + … + (*A*^*l*^)_i*,j*_ > 0 *thanks to the properties of the adjacency matrix*. *Of course, it makes no sense from a computational point of view to compute full powers of large adjacency matrices as it would be easier to compute from scratch the centrality measures. However, one can avoid this problem by using efficient methods for computing entries of the powers of A*. *For example, computing* (*A*^2^)_i*,j*_ *can be not too expensive as it can be obtained by the product of two vectors with less than* *d*_*max*_ ≪ *n* *nonzero entries, where d*_*max*_ *is the maximum degree among the node degrees*.

## 6 Numerical experiments

Here we report the results of experiments on synthetic and real-world networks, in which we compare our proposed strategies to simultaneous and sequential attacks based on TC. Synthetic networks (Poisson random graph and scale-free) are generated using erdrey and pref of the CONTEST MATLAB’s toolbox [29] with different parameters. The adjacency matrices of the real-world examples are available in the *SuiteSparse Matrix Collection* [28]. Among the synthetic networks, we consider only those connected while for real-world networks, the focus is only on the largest connected component. We remove also any self-loops from the graphs.

All the tests were performed on a laptop PC based on 2.38 GHz AMD Ryzen 4500U CPU with 16 GB RAM using MATLAB 2021a. Total communicability was computed using MATLAB expmv, which estimates *e*^*A*^**1** through a combination of the scaling and squaring method with a truncated Taylor series approximation. We made this choice since experimental comparisons with codes based on Krylov subspace show that expmv can sometimes perform better in terms of computational cost and accuracy in this particular task; see [30] for details.

In the experiments, we remove the top 1%, 10% and 20% nodes (according to TC) using all the strategies previously described. Then, we compared the rankings obtained using the intersection distance (2). In practice, we are measuring the distance between the orders of node elimination with a quantity, the intersection distance, that becomes closer to 1 as the discrepancy between the rankings increases.

Table 1 shows the results obtained for some synthetic networks with different sizes and degree distributions. First, from the value displayed in Table 1, we can observe that the distance between simultaneous and sequential attacks can be significative, even considering the cases in which only 1% of the nodes is removed. On the other hand, both threshold and correlation attacks seem to generally improve the results that can be obtained with simultaneous attacks in terms of approximation of sequential attacks. Specifically, threshold attacks provide the closest approximation even if the results appears to be really depend on the threshold value, that here we fixed at 0.01, suggesting that the threshold strategy may need some tuning to work at its best. In contrast, although the correlation attack strategy may appear less sharp than threshold attacks in many cases, it is important to mention that it does not require any external tuning.

**Table 1:** Intersection distances of sequential attacks with respect to simultaneous, threshold and correlation attacks in some synthetic networks.

		isim_1%_			isim_10%_			isim_20%_		
Network	n	sim.	thr.	cor.	sim.	thr.	cor.	sim.	thr.	cor.
erdrey(n,4n)	2000	0.1875	0.1235	0.1233	0.3512	0.1193	0.2435	0.3779	0.1336	0.2557
erdrey(n,4n)	6000	0.2135	0.1546	0.1679	0.3543	0.1283	0.2460	0.3745	0.1441	0.2683
erdrey(n,4n)	10000	0.2111	0.1434	0.1687	0.3560	0.1305	0.2501	0.3744	0.1413	0.2685
pref(n,2)	2000	0.3218	0.0122	0.1102	0.6471	0.0221	0.3384	0.6460	0.0213	0.3061
pref(n,2)	6000	0.5176	0.0193	0.1911	0.7193	0.0235	0.3763	0.6957	0.0255	0.4201
pref(n,2)	10000	0.5669	0.0207	0.2879	0.7446	0.0221	0.4329	0.7070	0.0259	0.4734

isim_*p*%_ shows the distance between the rankings obtained by removing the top *p*% of the nodes (according to TC). The values are the averages over 20 runs with the same parameters. The threshold value in threshold attacks is set to 0.01.

The results on threshold attacks in Table 1 suggest that Poisson random graphs may need a lower threshold to obtain a comparable decrease in the intersection distance with respect to scale-free networks. To further investigate this issue, we repeated our experiments on synthetic networks varying the threshold value. The results, shown in Table 2, confirm our claim and suggest that the inhomogeneous degree distribution of scale-free networks can cause more frequent computations of the centrality measures (and thus more precision in therms of approximation) due to the early elimination of high degree nodes.

**Table 2:** Intersection distances and computations using different threshold values in synthetic networks.

	pref(n,2)		erdrey(n,4n)	
Threshold	isim_10%_	TC computations	isim_10%_	TC computations
0.001	0	200	0.0026	191.95
0.005	0.0117	93.4	0.0833	39.95
0.01	0.0218	54.95	0.1278	20.75
0.05	0.0846	12.7	0.2496	4.95
0.1	0.1854	6.35	0.3193	2.25

isim_10%_ represents intersection distances between the rankings obtained by removing the top 10% nodes (according to TC) by sequential and threshold attacks, with various thresholds. The number of evaluations of centrality measures done by the threshold attack algorithm is also displayed. The values are the averages over 20 runs with the same parameters.

Regarding the efficiency of both the proposed strategies, Table 3 shows that it is possible to obtain a considerable reduction in the computational time when using our approach instead of sequential attacks for the considered centrality measure (and a similar effect is observed for other centrality measures based on matrix functions). This result suggests that our methods based on selective computation can represent a cheaper alternative compared to the sequential process of recomputing the measure after every node elimination in that framework.

**Table 3:** Computational time for sequential, threshold and correlation attacks in some synthetic networks.

		time 1%			time 10%			time 20%		
Network	n	seq.	thr.	cor.	seq.	thr.	cor.	seq.	thr.	cor.
erdrey(n,4n)	2000	0.064	0.010	0.017	0.598	0.064	0.122	1.119	0.117	0.227
erdrey(n,4n)	6000	0.399	0.023	0.078	3.486	0.145	0.65	6.218	0.240	1.146
erdrey(n,4n)	10000	1.546	0.050	0.197	13.187	0.297	1.585	22.266	0.496	2.860
pref(n,2)	2000	0.059	0.041	0.021	0.484	0.139	0.108	0.919	0.208	0.200
pref(n,2)	6000	0.323	0.124	0.076	2.376	0.146	0.436	4.301	0.411	0.803
pref(n,2)	10000	1.274	0.345	0.192	7.600	0.689	1.025	12.377	0.821	1.817

Computational time (in seconds) required to remove 1%, 10% and 20% of the most influential nodes (according to TC) for some synthetic networks produced with the CONTEST toolbox by sequential, threshold and correlation attacks. The values are the average of 20 runs with the same parameters. Here the threshold value in threshold attacks is set to 0.01. Times are based on MATLAB’s expmv.

**Fig 2:**
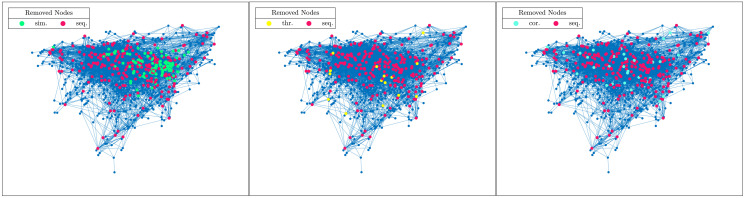
Comparisons between various strategies of attack, network *yeast*. Nodes removed from the network *yeast* in the case of simultaneous (in green), sequential (in red), threshold (in yellow) and correlation attacks (in light blue). The images are related to the cases in which the top 10% of the nodes (according to TC) are removed.

Turning now to the experiments on real-world networks, the results are consistent with the previous findings. Table 4 shows that threshold and correlation attacks approximate better sequential attacks than simultaneous as the values of the intersection distance are lower in all the cases considered. This result is even more clear if we look at the case of the network *yeast* displayed in Fig 2 in which we highlighted the rankings when the top 10% of the nodes (according to TC) are removed.

**Table 4:** Intersection distances of sequential attacks with respect to simultaneous, threshold and correlation attacks in some real-world networks.

		isim_1%_			isim_10%_			isim_20%_		
Network	n	sim.	thr.	cor.	sim.	thr.	cor.	sim.	thr.	cor.
Erdõs02	5534	0.3352	0.0737	0.1767	0.2292	0.0235	0.2068	0.2858	0.0162	0.3731
hep-th	8638	0.5522	0.0639	0.1198	0.6561	0.0833	0.1346	0.6148	0.0851	0.1229
Minnesota	2640	0.4894	0.4795	0.4085	0.5558	0.2612	0.4483	0.5465	0.1933	0.3998
Power	4941	0.6437	0.1468	0.2796	0.6356	0.0758	0.2714	0.6104	0.0596	0.2653
as-735	6474	0.3298	0.0099	0.0492	0.6609	0.0150	0.1944	0.7106	0.0326	0.2243
yeast	2361	0.4263	0.0419	0.0935	0.5681	0.0810	0.1329	0.5110	0.0668	0.1399
as-22july06	22963	0.3367	0.0196	0.1403	0.6156	0.0198	0.4498	0.6434	0.0654	0.0654
usroads-48	126146	0.6677	0.6116	0.4178	0.6393	0.3579	0.3769	0.6143	0.2648	0.2648

isim_*p*%_ represents the distance between the rankings obtained by removing the top *p*% of the nodes (according to TC). The threshold value in threshold attacks is set to 0.01.

Regarding the comparison between threshold and correlation attacks, we can observe that the first strategy seem more efficient and accurate in most cases whenever a good threshold is known or computed, e.g., from a-priori known data. However, the fact that correlation attacks do not need to fix a threshold value is particularly interesting in the case of medium/large networks like *usroads-48*, where the choice of the threshold has a substantial impact on the results. To be more precise, Table 4 show that for scale-free networks such as *as-735* and *as-22july06*, it is possible to obtain smaller values of the intersection distance with threshold attacks in comparison to what happens for networks similar to a Poisson random graph such as *Minnesota*, *Power* and *usroads-48*.

Finally, Table 5 shows that our strategies ensures far better results in terms of efficiency even on real-world networks. This conclusion is especially relevant in the case of large networks like *as-22july06* and *usroads-48*, where is possible to achieve a highly accurate approximation of the rankings obtained by sequential attacks with a fraction of the computational cost.

**Table 5:** Computational time for sequential, threshold and correlation attacks in some real-world networks.

		time 1%			time 10%			time 20%		
Network	n	seq.	thr.	cor.	seq.	thr.	cor.	seq.	thr.	cor.
Erdõs02	5534	0.285	0.105	0.113	1.771	0.426	0.588	2.832	0.441	1.001
hep-th	8638	2.532	1.093	1.374	10.413	1.868	3.497	14.905	2.099	5.011
Minnesota	2640	0.067	0.009	0.030	0.626	0.050	0.218	1.196	0.098	0.415
Power	4941	0.187	0.044	0.087	1.588	0.144	0.579	2.941	0.222	1.070
as-735	6474	0.549	0.364	0.283	2.387	0.648	0.905	3.750	0.702	1.455
yeast	2361	0.091	0.046	0.049	0.673	0.217	0.268	1.150	0.335	0.432
as-22july06	22963	7.926	2.321	2.729	24.124	3.210	7.935	32.128	3.313	12.648
usroads-48	126146	91.007	0.230	28.404	778.975	1.419	274.61	1359.232	2.510	462.31

Computational time (in seconds) required to remove 1%, 10% and 20% of the most influential nodes (according to TC) for some real-world networks by sequential, threshold and correlation attacks. Here the threshold value in threshold attacks is set to 0.01. Times are based on MATLAB’s expmv.

## 7 An application to robustness

Let us now consider the application of the strategies developed for the selective computation of certain centrality indices to the robustness of a complex network.

In the analysis of complex networks, the notion of robustness refers to the ability of a system to resist to the failures of some of its components, such as a group of nodes or a subset of arcs. Here we assume, as suggested in [19], that it is possible to focus exclusively on the effects of node and edge removal on the network structure, mainly on the size of its largest connected component. If the latter becomes small, it is reasonable to think that the system and its communication capabilities could be compromised.

Let *G*_*ρ*_ be the graph obtained following the elimination of a fraction of nodes *ρ* from the graph *G*, which has *N* nodes. If we indicate with $G_{\rho }^{\prime }$ the largest connected component of *G*_*ρ*_, it is possible to define the function *σ*(*ρ*) as the ratio of the number of nodes in $G_{\rho }^{\prime }$ and *N*:

$$\begin{array}{c} \sigma \left(\rho \right)=\frac{|G_{\rho }^{\prime }|}{N}. \end{array}$$ (4)

As previously observed in [9], computing the values of *σ*(*ρ*) allows to quantify the graph response to the targeted removal of a fraction *ρ* of its nodes.

Once a method has been chosen for ranking the nodes, one can proceed computing *σ*(*ρ*) by progressively increasing the fraction of nodes that are subtracted from the network, by choosing, at each step, the node with the highest centrality. In this way, it is possible to define the robustness of a network with respect to targeted attacks, i.e. its *R-index* [31]:

$$R=\frac{1}{N}\sum_{i=1}^{N}\sigma \left(i/N\right),$$ (5)

where the normalization factor 1/*N* enables comparisons among the robustness with networks of different sizes. The minimum value that the quantity *R* can take is 1/*N* and it is obtained for star graphs. On the other hand, for complete graphs, we get the maximum value of *R*, that is (1/2)(1 − 1/*N*). Consequently, given that for each type of graph $R\in [0,\frac{1}{2}]$, it is possible to define an index *V* complementary to *R* as the quantity:

$$\begin{array}{c} V=\frac{1}{2}-R. \end{array}$$ (6)

This index is called *V-index* and is a measure of a network’s vulnerability to targeted attacks.

Clearly, the changes of *V* (and *R*) depend on the selected method to rank the nodes. Moreover, if a centrality measure is used, there is a substantial difference between recomputing and not recomputing the measures after a node elimination. As observed in [9], networks generally exhibit wider vulnerability to sequential attacks regardless of the type of centrality index considered. This is in agreement with the assumption that establishing a ranking dynamically, by recalculating the centrality, is more effective in identifying the weak points of a complex network.

**Fig 3:**
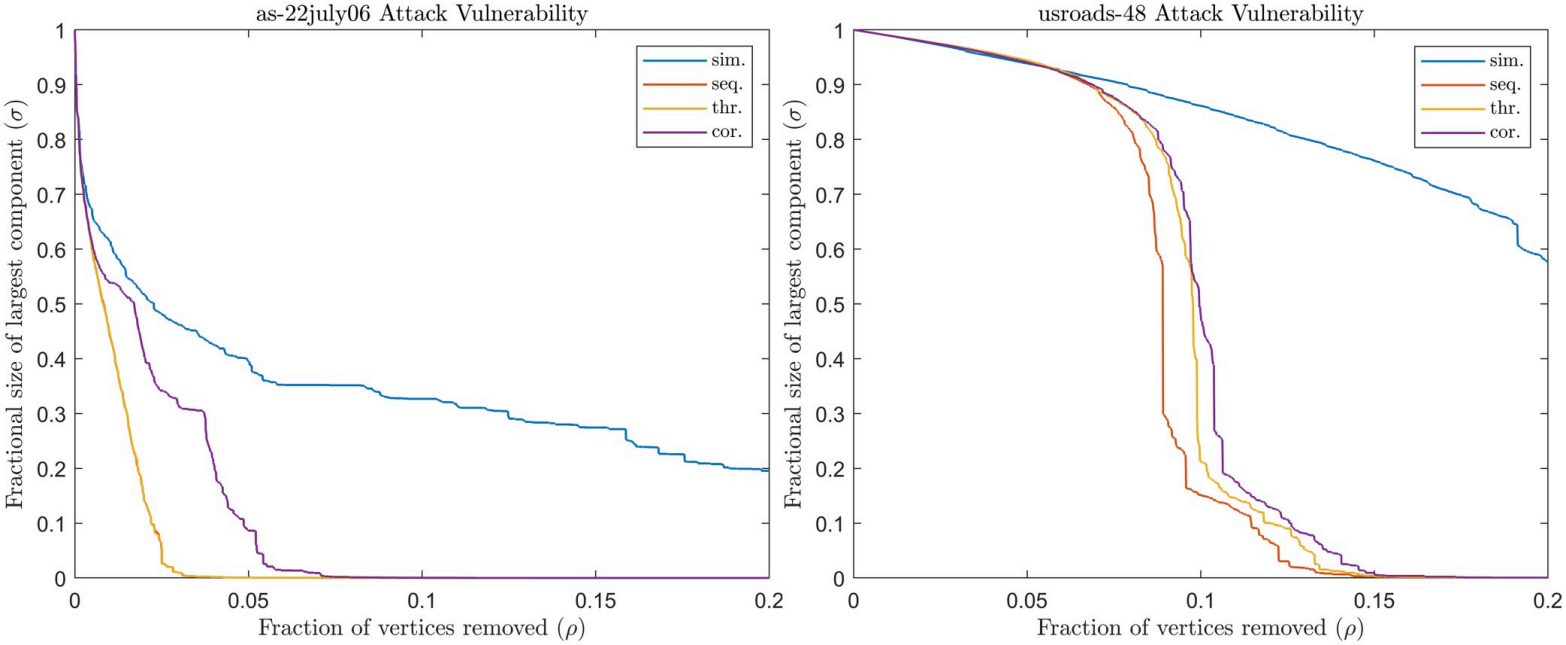
Vulnerability for simultaneous, threshold and correlation attacks. Network as-22july06 and usroads-48.

As a consequence of this observation, we are naturally more interested in evaluating the robustness and vulnerability of a network against sequential attacks rather than against simultaneous attacks, despite the significant computational resources required to do so. However, we can use our proposed techniques based on selective computation to efficiently approximate the sequential attack vulnerability index. Table 6 shows the results of our experiments on the vulnerability of some synthetic and real-world networks to various attack strategies based on TC. From the data reported, we can confirm that threshold and correlation attacks can be considered interesting and cheaper alternative to sequential attacks in the study of vulnerability for large networks for some centrality indices. Moreover, the *σ*(*ρ*) plots in Fig 3 show that the proposed strategies can also be used to estimate the critical percentage of nodes that should be removed with sequential attacks to disconnect the largest connected component; i.e. such that *σ*(*ρ*) = 0.

**Table 6:** Vulnerability indices for some synthetic and real-world networks.

Network	*V*_*sim*._	*V*_*thr*._	*V*_*cor*._	*V*_*seq*._
pref(n,2)	0.1827	**0.4084**	0.3330	0.4093
erdrey(n,4n)	0.0561	**0.1517**	0.1216	0.1532
Erdõs02	0.4670	**0.4739**	0.4709	0.4740
hep-th	0.2277	0.3722	**0.3744**	0.3757
Minnesota	0.3454	**0.4075**	0.3848	0.4111
Power	0.3619	**0.4489**	0.4421	0.4502
as-735	0.3388	**0.4876**	0.4867	0.4877
yeast	0.2746	**0.3872**	0.3778	0.3883
as-22july06	0.4003	**0.4901**	0.4802	0.4901
usroads-48	0.3031	**0.4059**	0.4028	0.4126

The indices are computed by simulating simultaneous, sequential, threshold and correlation attacks based on TC. The values for synthetic networks are averaged over 20 runs with *n* = 10000 using the MATLAB’s CONTEST toolbox. The threshold value in threshold attacks is set to 0.01.

## 8 Final remarks, directions and conclusions

We investigated the problem of updating centrality indices in a network after node or arc removal. We discussed two well-known strategies of simultaneous and sequential attacks showing how much those can differ in terms of efficacy and computational cost when considering centrality indices based on matrix functions. Moreover, we proposed two strategies based on selective computation, threshold and correlation attacks, that aim to overcome the inaccuracy of simultaneous attacks and the potentially higher computational cost of sequential attacks.

Numerical experiments showed that the proposed strategies are prone to be less expensive in terms of computational time than sequential attacks while potentially maintaining a better approximation than simultaneous attacks for certain centrality indices, in particular for the ones that are based on matrix functions. Experiments on robustness indicated that our strategies could also be considered cheap alternatives to approximately compute some sequential attacks vulnerability indices.

We stress that, even if our techniques are developed focusing on walk-based centrality indices, they are applicable in principle to every measure of centrality, in particular for threshold attacks.

In a further study, we plan to concentrate on the computational efficiency and feasibility of threshold and correlation attacks considering also *update attacks*, a new strategy based on a hybrid update of some centrality indices computed by functions of matrices. We also plan to investigate attacks related to network dynamics by using some of the tools generalized in [32, 33].
